# Exceptional longevity is associated with decreased reproduction

**DOI:** 10.18632/aging.100415

**Published:** 2011-12-26

**Authors:** Vafa Tabatabaie, Gil Atzmon, Swapnil N. Rajpathak, Ruth Freeman, Nir Barzilai, Jill Crandall

**Affiliations:** Institute for Aging Research, and Division of Endocrinology, Albert Einstein College of Medicine, Bronx, NY 10461, USA

**Keywords:** longevity, fertility, centenarian, reproduction, pregnancy, aging

## Abstract

A number of leading theories of aging, namely The Antagonistic Pleiotropy Theory (Williams, 1957), The Disposable Soma Theory (Kirkwood, 1977) and most recently The Reproductive-Cell Cycle Theory (Bowen and Atwood, 2004, 2010) suggest a tradeoff between longevity and reproduction. While there has been an abundance of data linking longevity with reduced fertility in lower life forms, human data have been conflicting. We assessed this tradeoff in a cohort of genetically and socially homogenous Ashkenazi Jewish centenarians (average age ~100 years). As compared with an Ashkenazi cohort without exceptional longevity, our centenarians had fewer children (2.01 vs 2.53, p < 0.0001), were older at first childbirth (28.0 vs 25.6, p < 0.0001), and at last childbirth (32.4 vs 30.3, p < 0.0001). The smaller number of children was observed for male and female centenarians alike. The lower number of children in both genders together with the pattern of delayed reproductive maturity is suggestive of constitutional factors that might enhance human life span at the expense of reduced reproductive ability.

## INTRODUCTION

The relationship between longevity and fertility has been extensively investigated, both in human and in lower life forms, but has produced conflicting results. While most human studies have suggested a trade off between longevity and fertility [1-4], others have reported a positive correlation [5-7] or no consistent association [8-11]. These conflicting results could be partly attributed to unsuitable control groups, such as comparing individuals from different birth cohorts or to differences in socioeconomic status of study populations, incomplete data collection in historic cohorts and other methodological issues [12].

We sought to address this question in a case-control study of a unique community-based cohort of Ashkenazi Jewish individuals with exceptional longevity. Today, Ashkenazi Jews comprise about 80 percent of the Jews in the United States and are an attractive target for genetic studies of aging and mechanisms of disease due to their relative genetic homogeneity and sizable numbers.

## RESULTS

### Participants

The study population consisted of People with Exceptional Longevity defined as those who have reached a minimum age of 95 (PEL; n = 525; 75% females). PEL were born around the turn of the century and reached reproductive age in the 1920's. We generated a control group, non-PEL, by collecting life data from an unrelated group of elderly Ashkenazi Jewish individuals (spouses or friends of PEL offspring), determining their parents' reproduction history. Non-PEL were therefore contemporaneous with PEL, but died before age 95 (non-PEL; n = 193; mean age at death for women 74.9 (SD 14.5); mean age at death for men 72.4 (SD 13.4); range 26-94 years).

### PEL have fewer children than non-PEL

**Figure 1:**
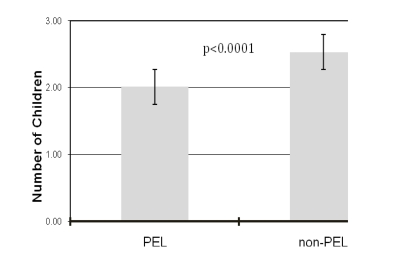
The number of children born to people with exceptional longevity (PEL) and a non-PEL control group.

In our cohort, PEL had significantly fewer children than non-PEL (2.01 vs 2.53; p < 0.0001) (Figure 1). This lower parity among PEL was not related to gender, since both female and male PEL individuals had lower number of children as compared to non-PEL (number of children for female PEL = 1.97 vs male PEL = 2.16; p = 0.08). Similarly, among PEL, the number of children did not differ by level of education (number of children for PEL with high school diploma or below = 1.92 vs. PEL with higher education = 1.82; p = 0.4).

### PEL exhibit delayed fertility

We found additional support for the concept of delayed reproductive maturity in our cohort. Since reliable data about age at first and last pregnancy and menarche were not available for non-PEL, we compared these values in PEL and the offspring of the non-PEL control group. PEL were significantly older at menarche (13.2 vs 12.3; p < 0.0001), at birth of their first child (28.0 vs. 25.6; p < 0.0001) as well as last child (32.4 vs. 30.3; p < 0.0001).

## DISCUSSION

Our results support the notion of a tradeoff between longevity and reproduction in humans. PEL and non-PEL individuals in our cohort were all born around the turn of the century and reached reproductive age in the 1920's, long before reliable methods of birth control were widely available. This reduces the influence of contraception on family size, a potential caveat in similar studies on more contemporary populations.

The disposable soma theory of aging argues that investment in reproduction deprives organisms of resources required for self-maintenance, thus reducing longevity. This effect might be more easily explained in females through direct physical burdens of pregnancy, childbirth and breast-feeding. However, in our PEL cohort we observed similar reduced fertility among male and female participants, suggesting that factors other than mere physical stressor of reproduction (e.g. unrecognized genetic factors that affect males and females equally) may also be responsible. Support for this observation can be found in the Reproductive-Cell Theory of Aging, which maintains that hormones that promote growth and development early in life to achieve reproductive maturity act in an antagonistic pleitropic manner later, promoting senescence [13, 14]. A genetic predisposition to hinder and/or delay these hormonal mechanism might reduce reproductive success and at the same time delay aging and mortality in women and men alike [2, 15]. A potential physiologic mechanism may be related to genes involved in cholesterol metabolism. Since we find a favorable cholesterol phenotype and genotype in our subjects (CETP, APOC3, ADIPOQ) [16, 17] it is possible that such genes delay gonadal maturity in the young while protecting them later in life from adverse phenotypes commonly associated with aging, thus extending life span.

In order to explain the trade-off between longevity and reproductive ability, other investigators have focused on the possible role of the mTOR signaling pathway [18, 19]. The mammalian target of rapamycine (mTOR) is responsible for sensing cellular energy status and for coupling it to cell growth and proliferation [20]; it is also believed play a role in central regulation of puberty. Acute activation of mTOR in pubertal female rats stimulates LH secretion, whereas blockade of central mTOR signaling by rapamycin inhibits gonadotropic axis and delays puberty [21]. Others have shown that inhibition of mTOR extends life span of invertebrates [22] and mammals [23]. Therefore it seems that a weak mTOR signaling pathway might lead to longevity at the expense of delayed puberty or limited fertility.

These results confirm the observation that women who achieve exceptional longevity reproduce later in life, as also reported by others [2, 3, 5-8, 15]. PEL were 2.5 years older than the offspring of non-PEL at their first childbirth and 2.1 years older at last childbirth. We find these results especially significant, since this comparison was done between females of two consecutive generations. Although various historic variables like war and the Great Depression might have contributed to delayed pregnancy in PEL, overall the mean age at first childbirth has steadily increased in the past 30 years, according to data from National Vital Statistics Report, CDC. It has been suggested that the ability to have children in fifth decade of life may be a marker for slow aging and subsequent longevity [7, 15]. Alternatively, it might be a marker of genetically delayed or limited fertility in centenarians, also suggested by others [2, 15]. On the other hand, earlier menarche in non-PEL offspring can also be explained by improved nutrition in later generations.

The strengths of our study include a well-characterized and homogenous study population, comparison within the same birth cohort and large sample size. In addition, direct collection of information about family size from PEL subjects and from children of non-PEL reduces the likelihood of underreporting number of children, a common critique of similar studies done on more historic populations who might have underreported the number of children who died young or the number of female children [2, 7, 12]. However, certain limitations of our study warrant consideration. It is unclear whether these results can be generalized to other ethnicities, although survival and cause of death in Ashkenazi Jews are similar to those of the general white population in the US [16]. Also, the observational nature of our study precludes any causal inference.

In conclusion, our study shows that individuals who achieve exceptional longevity have fewer children than a contemporaneous population with usual survival, and that they tend to reproduce later in life. Further studies are needed to confirm this finding and to establish the mechanisms responsible for this delayed and reduced reproductive ability.

## METHODS

Recruitment methods for the Searching for Longevity Genes in the Historically Unique Ashkenazi Jewish Population Study and characteristics of the cohort have been described in detail elsewhere [16, 17, 24, 25]. Briefly, subjects were recruited by word of mouth, through advertisements in Jewish aging centers and homes, and through publicity in synagogues and Jewish media, mostly in the New York area. A trained interviewer collected data on socio-demographics and family structure, number of children including those who died from all consenting members of the families. In case of PEL, stated ages were verified by checking participants' passports or birth certificates. In case of non-PEL, age at time of death and number of children were collected through interviewing their children. In many instances, multiple members of the same family took part in the study, providing additional validation of parental life span, family size and other data.

We excluded PEL who married late in life or if the duration of their marriage was too short for completing their family, although only 2 individuals had to be excluded due to these criteria. We used student's t-test to compare mean number of children and age at first and last pregnancy. Statistical analysis was conducted using SPSS (Chicago, IL) and p-values less than 0.05 were considered statistically significant.
